# Distributed Wearable Ultrasound Sensors Predict Isometric Ground Reaction Force

**DOI:** 10.3390/s24155023

**Published:** 2024-08-03

**Authors:** Erica L. King, Shriniwas Patwardhan, Ahmed Bashatah, Meghan Magee, Margaret T. Jones, Qi Wei, Siddhartha Sikdar, Parag V. Chitnis

**Affiliations:** 1 Department of Bioengineering, George Mason University, Fairfax, VA 22030, USA; shriniwas.patwardhan@nih.gov (S.P.); abashat2@gmu.edu (A.B.); qwei2@gmu.edu (Q.W.); ssikdar@gmu.edu (S.S.); 2 Center for Adaptive Systems of Brain-Body Interactions, George Mason University, Fairfax, VA 22030, USA; 3 Frank Pettrone Center for Sports Performance, George Mason University, Fairfax, VA 22030, USA; mjones15@gmu.edu; 4 National Institute of Health, Bethesda, MD 20892, USA; 5 School of Kinesiology, George Mason University, Fairfax, VA 22030, USA; mmagee3@kent.edu; 6 School of Sports, Recreation and Tourism Management, George Mason University, Fairfax, VA 22030, USA; 7 School of Health Sciences, Kent State University, Kent, OH 44240, USA

**Keywords:** wearable ultrasound, force production, neuromuscular monitoring

## Abstract

Rehabilitation from musculoskeletal injuries focuses on reestablishing and monitoring muscle activation patterns to accurately produce force. The aim of this study is to explore the use of a novel low-powered wearable distributed Simultaneous Musculoskeletal Assessment with Real-Time Ultrasound (SMART-US) device to predict force during an isometric squat task. Participants (N = 5) performed maximum isometric squats under two medical imaging techniques; clinical musculoskeletal motion mode (m-mode) ultrasound on the dominant vastus lateralis and SMART-US sensors placed on the rectus femoris, vastus lateralis, medial hamstring, and vastus medialis. Ultrasound features were extracted, and a linear ridge regression model was used to predict ground reaction force. The performance of ultrasound features to predict measured force was tested using either the Clinical M-mode, SMART-US sensors on the vastus lateralis (SMART-US: VL), rectus femoris (SMART-US: RF), medial hamstring (SMART-US: MH), and vastus medialis (SMART-US: VMO) or utilized all four SMART-US sensors (Distributed SMART-US). Model training showed that the Clinical M-mode and the Distributed SMART-US model were both significantly different from the SMART-US: VL, SMART-US: MH, SMART-US: RF, and SMART-US: VMO models (*p* < 0.05). Model validation showed that the Distributed SMART-US model had an R^2^ of 0.80 ± 0.04 and was significantly different from SMART-US: VL but not from the Clinical M-mode model. In conclusion, a novel wearable distributed SMART-US system can predict ground reaction force using machine learning, demonstrating the feasibility of wearable ultrasound imaging for ground reaction force estimation.

## 1. Introduction

Return to sport following a musculoskeletal injury focuses on reestablishing normal biomechanical patterns and, if done correctly, decreases reinjury risk by 85% [1]. When performing tasks that require movement, muscles work together in tandem to execute the movement and produce the required force [2]. Current rehabilitation techniques rely upon visual assessments and palpations during slow controlled movements as well as patient reports to monitor the recovery of muscle function [3]. Thus, current exams are subjective and heavily dependent upon the clinician’s experience and patient feedback. To achieve more quantitative assessment, state-of-the-art return to sport protocols utilize kinetic measures involving the utilization of sensors such as force plates, dynamometers, and 3D motion capture [4,5]. These are extensions of routine clinical assessments; however, these measures require large equipment, specialized personnel, post hoc analysis, and are generally not found outside of biomechanical laboratories.

Force plates have become a main component for monitoring sports performance testing and neuromuscular fatigue [4,6,7,8,9]. The increased portability of force plates in recent years has made them standard practice in the sports performance field [10,11]. Specifically, isometric squat testing is a monitoring tool commonly used to assess the rate of force development in athletic populations [12,13,14]. Previous literature has demonstrated a strong correlation between isometric squat testing and other dynamic performance tasks (e.g., jump testing) [13,15].

However, force plates only consider ground reaction forces that are produced from multiple muscle groups working together. How individual muscles are activated and contribute to force production cannot be determined from the use of force plates. This level of information is needed during return to sport protocols to ensure that an athlete is not compensating during a functional task, resulting in improper form or inefficient movement [16,17].

Muscle-level information can be acquired using surface electromyography (sEMG) to monitor activation patterns during dynamic movement [4,18,19]. However, sEMG has limitations, including complex outputs, noise influences, muscle cross-talk, and the inability to derive signals from deep muscle tissue [20,21]. Mechanomyography (MMG), a technique to measure mechanical activity of the muscle using specific transducers, can also be used to obtain muscle-level information [22,23,24]. Previous research has shown that MMG can monitor muscle function during activity but is limited, as signals are influenced by movement artifacts [25]. Together, sEMG and MMG have been combined to assess muscle function information during dynamic tasks [22,25,26].

Ultrasound imaging is an attractive method to image and characterize muscle tissues [27,28]. However, current ultrasound-based examinations are not dynamic and are performed subjectively in highly controlled environments where the patient is stationary. Dynamic ultrasound imaging allows one to characterize muscle contraction, velocity, pennation angle, and fascicle length changes, which can be inform muscle function [29,30,31,32,33]. Kamatham et al. have shown the application of ultrasound metrics in predicting muscle force during isometric hand contractions [34]. Additional studies have also shown changes in ultrasound measures, such as pennation angle and echogenicity, over the duration of long periods of intense exercise [35]. 

Although the aforementioned studies have shown promising results, implementation in clinical settings is challenging as current clinical systems are not optimized for dynamic imaging. Furthermore, clinical ultrasound systems are hindered by bulky transducers that require hand-held operation or custom strapping mechanisms to maintain acoustic coupling during dynamic movement, typically from a single anatomical site. In such settings, even the slightest transducer movement can cause skin contact loss and/or changes in the transducer pressure, resulting in inaccurate muscle function characterization [36]. To be feasible for imaging in dynamic and clinical settings, ultrasound systems must be able to overcome these limitations. 

Considering the challenges of acquiring dynamic ultrasound images using traditional ultrasound imaging transducers, wearable ultrasound technology research and development has accelerated in recent years. Current applications of wearable ultrasound technology have mainly focused on cardiovascular and pulmonary function, exoskeleton, and prosthesis control [37,38,39,40,41,42,43]. Wearable ultrasound holds great promise for simultaneously imaging different muscle groups contributing to force production during movement to assess muscle function in a comprehensive and systematic fashion. However, this requires the development of a miniaturized system enabling distributed imaging of multiple muscle groups simultaneously. 

Researchers have previously shown that the use of motion mode (M-mode) ultrasound imaging can be a viable alternative to traditional B-mode imaging in investigating time-varying muscle deformation [44]. M-mode involves imaging a single ultrasound scanline over time, allowing muscle motion to be monitored more efficiently [33,45]. Utilizing M-mode imaging allows for simpler electronics, smaller device footprints, lower computational requirements and the incorporation of multiple imaging sensors. 

We have developed a Simultaneous Musculoskeletal Assessment with Real Time Ultrasound (SMART-US) device that uses novel ultrasound imaging technology to acquire four channels of ultrasound M-mode data concurrently [46,47,48]. In this study, our research objective was to investigate whether ground reaction force during an isometric squat task can be predicted from M-mode ultrasound data from our novel distributed system using a machine learning algorithm. The distributed nature of SMART-US means that different sensors target different muscles simultaneously to get a more holistic view of muscle physiology and synergistic activation of different muscles during physical activity. This system differs from other multi-channel systems, which place all the sensors on one muscle similar to [49] or spread them out in a circular manner around the limb to mitigate chances of collecting low-quality data for processing algorithms [37]. The three following hypotheses were tested: (1) M-mode ultrasound imaging can predict ground reaction force during isometric squats, (2) force predictions using distributed SMART-US sensor are comparable to those obtained from traditional clinical ultrasound systems, and (3) a distributed SMART-US imaging device will have higher accuracy than a single-channel image acquisition system. Our results indicate support for all these three hypotheses.

## 2. Materials and Methods

Five able-bodied female subjects (24 ± 3.4 years) participated in the study. All participants were former National Collegiate Athletic Association Division I athletes, in good physical health, and recreationally active. They reported no lower body injuries, had the risks and benefits explained before testing, and signed an institutionally approved informed consent form. 

Before testing began, the dominant leg was determined by asking the participants which leg they used to kick a ball [50]. The participants’ dominant leg was then scanned with a clinical ultrasound transducer (eSaote BioSound MyLab 25, Biosound Esaote, Inc., Indianapolis, IN, USA). Static B-mode images were acquired at four locations: Rectus Femoris, Vastus Lateralis, Medial Hamstring, and Vastus Medialis. Locations were standardized across all participants by finding the midpoint of the upper leg measuring from the Anterior Superior Iliac Spine (ASIS) to the midpoint of the lateral knee. The midpoint of the upper leg was marked across the upper leg and muscle palpation and static m-mode ultrasound scans were used to identify and confirm muscle locations. Locations of clinical images across the upper leg were marked using a custom template that outlined the shape clinical scanner and had a midline mark to show were the wearable sensor placement were to be placed later in the protocol. Due to the large size of the upper leg muscles, even if there was slight variation in sensor placement, results would still be reliable, as the sensor would still be over the muscle of interest. After scanning was concluded, participants completed a standardized 15-min dynamic exercise warm-up. 

Following the warm-up, participant conducted two rounds of three isometric squats at maximum voluntary contraction on a force plate (AccuPower; AMTI, Watertown, MA, USA) acquired at 1600 Hz with a custom data acquisition system utilizing LabVIEW (National Instruments, Austin, TX, USA). Ground reaction force was reported in Newtons. Two rounds of non-randomized ultrasound imaging conditions were conducted with at least 10 min of rest in between rounds. Participants were cued using verbal commands from researchers on when to start and end the movement. Participants executed a maximum voluntary contraction squat for approximately 5 s when cued. All data were time-synched using a custom keyboard that enabled the researchers to start and stop the data collection on each machine simultaneously.

### 2.1. Round 1—Clinical Ultrasound Testing

**Figure 1:**
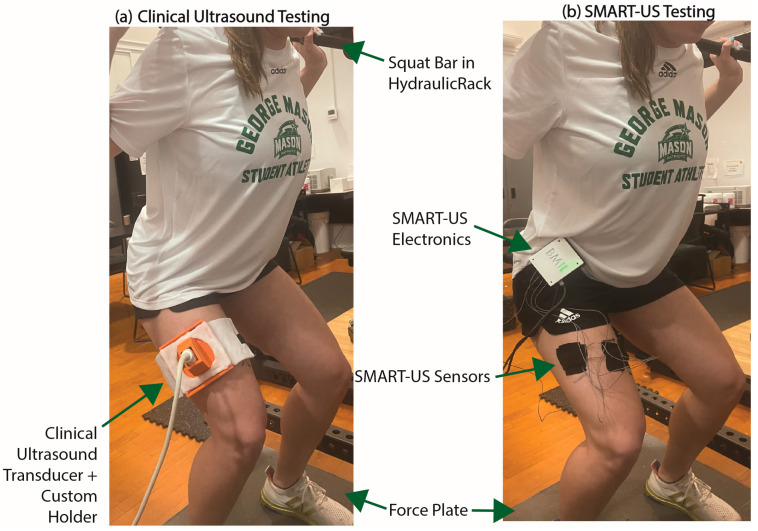
(**a**) Participant in an isometric squat on a force plate with a clinical MSK-US transducer fixed to their dominant vastus lateralis with a custom 3D-printed holder and strapping mechanism. (**b**) Participant in an isometric squat on a force plate with SMART-US device fixed to the waistband of their shorts. SMART-US electronics are set in a custom 3D-printed housing and four PZT transducers are located on the Vastus Lateralis, Rectus Femoris, Medial Hamstring, and Vastus Medialis and fixed via kinesiology tape.

A clinical two-dimensional ultrasound system (eSaote BioSound MyLab 25, Biosound Esaote, Inc., Indianapolis, IN, USA) with a 5-cm linear transducer (frequency, 7.5 MHz; axial resolution < 0.5 mm) was used for round one of testing. The clinical system was fitted to the participant’s dominant vastus lateralis using a custom 3D-printed holder (Figure 1a). The vastus lateralis was chosen for this task because it one of the main muscle that contribute to force production during a squat task [51]. Additional clinical ultrasound testing protocols at different anatomical locations were not included to limit participant fatigue.

Images were collected using a USB-based video grabber (DVI2USB 3.0, Epiphan Systems, Inc., Palo Alto, CA, USA) at 50 Hz during the isometric squat. Ultrasound settings remained constant across participants (Depth: 4 cm, Gain: 50).

**Figure 2:**
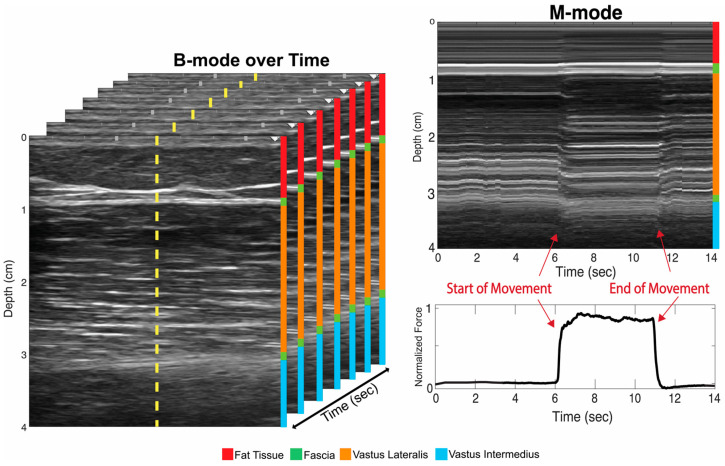
A set of clinical B-mode ultrasound images taken over time with a single scan line (yellow). On the left, an M-mode image created from the yellow scanline on the B-mode stack on the right over time. The start of the movement and end of movement is labeled (red arrows) as the M-mode in relation to the force trace.

After data acquisition, the two-dimensional ultrasound image sets were imported into a custom MATLAB (MATLAB 2022b, MathWorks Inc., Natick, MA, USA) script and transformed into an M-mode image. This was accomplished by taking a singular scanline at the center of the two-dimensional image over time (Figure 2).

### 2.2. Round 2—SMART-US Testing

A wearable, custom designed ultrasound system with multiple distributed imaging sensors, was strapped to the participant’s waist (Figure 1b). Additional device details can be found in previous publications [48] but a summary of the capabilities is included in this section. The SMART-US system can capture four channels of M-modes at a frame rate of 50 Hz and a depth of 4 cm using PZT transducers with a center frequency of 4.25 MHz. The system uses a frequency sweep with known sweep rate (10 ms long) to encode depth as frequency, such that shallow depths are low frequency components and deeper muscles are higher frequency components in a multi-component sinusoidal signal. Image processing consists of performing a fast Fourier transform (FFT) on the 10 ms window to acquire the frequency components and thus the muscle interfaces at where the transducers were placed. Transducers were placed in custom 3D-printed housings on the dominant leg in the following locations: vastus lateralis (at the center of the clinical transducer from round one), rectus femoris, medial hamstring, and vastus medialis (Figure 1b). These specific muscles for SMART-US sensor placement were selected because they have been shown to produce the most activation during a squat task [51]. Sensor placement for the vastus lateralis was determined using a custom 3D-printed template, which marked the center of the clinical ultrasound transducer, ensuring placement was the same across collections. 

### 2.3. Modeling

A ridge regression model was used for machine learning analysis to predict ground reaction force in Newtons from ultrasound image features. Ridge regression estimates coefficients of linear models that include a linearly correlated predictor. Specifically, this model is used to address the problem of multicollinearity by utilizing a penalty term:

(1) $$\beta ={\left(X^{T}X+kl\right)}^{-1}X^{T}y$$

where *k* is the ridge parameter *(k* = 5), and *l* is the identity matrix. Smaller positive values of *k* improve the conditioning and reduce the variance of model estimates, resulting in a smaller mean squared error [52]. 

The ridge function used in MATLAB solves the following equation for a given λ value:

(2) $${min}_{\beta_{0},\beta }\left(\sum_{i=1}^{N}{\left(y_{i}-\beta_{0}-x_{i}^{T}\beta \right)}^{2}+\sum_{j=1}^{p}\beta_{j}^{2}\right)$$

where *N* is the number of observations, *y_i_* is the response at observation *i*, *x*_i_ signifies the data with a vector of length *p* at observation *i*, λ is a nonnegative regularization parameter, $\beta_{0}$ is a scalar, and β is a vector the length of *p*.

#### 2.3.1. Feature Extraction

Image features were extracted from M-mode images using a mean depth calculation. Mean depth was used to track the muscle interface as it expands or contracts during the specified task. The calculation is a weighted average, that weighs each pixel by the pixel intensity at that corresponding depth. This, in turn, results in a frequency, and in this case depth, where most of the energy is centered in the window. Feature sections are calculated by taking a moving depth window of 25 pixels over each amplitude line (A-line) of the image, with a window overlap of 50%. To calculate each window’s mean depth, the window’s pixel intensities (*A*) are first multiplied by the corresponding pixel depth (*D*). The sum of the multiplied components is then divided by the sum of the pixel intensities in that window (Equation (3)).

(3) $$Mean\;Depth=\frac{\sum_{i=1}^{n}A_{i}D_{i}}{\sum_{i=1}^{n}A_{i}}$$

**Figure 3:**
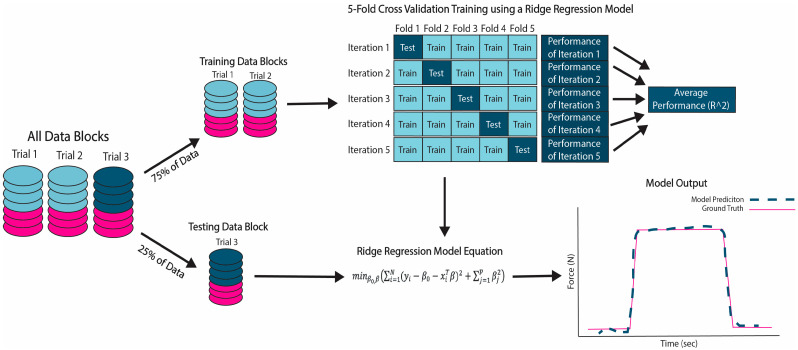
Schematic of ridge regression models for training and testing. Data were compiled (blue = image data, pink = force data) for each trail and then split into training blocks (Trials 1 and 2) and testing blocks (Trial 3) for each subject). A 5-fold cross-validation was used for training the ridge regression model and performance was reported as R^2^. Testing of the model was performed utilizing the Trial 3. The model output shows the ground truth force (N) (solid pink line) compared to the model prediction force (N) (dashed blue line).

The extracted features of each column in the M-mode image were then converted to Z-scores. A principal component analysis (PCA) was run the resulting Z-scores to obtain the most significant features. The features that explained 99% of the variance were retained for further analysis. Data containing all features were split into training and testing sets to validate and test the machine learning models (Figure 3). The acquired M-mode images were put into six categories for analysis and comparison (Table 1)

#### 2.3.2. Model Validation

**Figure 4:**
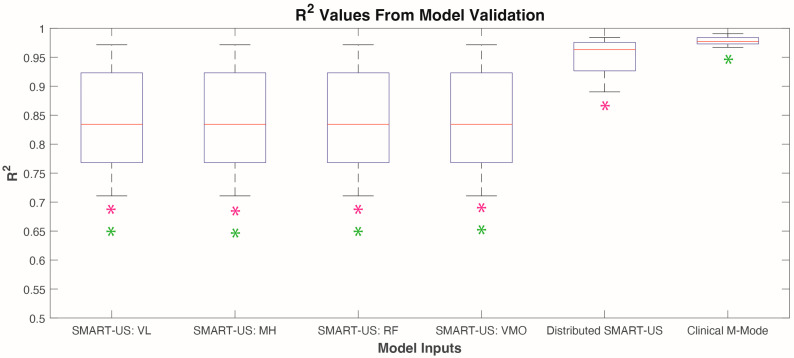
Results of cross-validations of six models (n = 5 participants). * *p* < 0.05. Pink * denotes significant differences between the Distributed SMART-US model and other models. Green * denotes significant differences between the Clinical M-mode model and other models.

Trials 1 and 2 were used for 5-fold cross-validation to evaluate the model’s performance in making predictions compared to the ground truth, which is force plate data obtained during the movement. Each round of cross-validation involves randomly partitioning all feature data into five subsets of equal size, with four subsets used for training and the remaining for testing. The process is repeated five times to ensure that each subset is used once as the testing set with the other subsets used to train the model. The participants average cross-validation error, reported as R^2^, was used to evaluate performance (Figure 4).

#### 2.3.3. Model Testing

The third trials were reserved for model testing. The predicted force was filtered before the R^2^ calculation using a moving averaged filter with a window size of 25 pixels. The R^2^ value for each tested trial was calculated and reported.

### 2.4. Statistical Analysis

The coefficient of determination (R^2^) was the primary outcome measure used to evaluate model performance and determine the accuracy of the predicted force outputs compared to the ground-truth force measures. An R^2^ coefficient closer to 1 was indicative of a higher model accuracy. 

Data were assessed for normality via Levene’s test. If data did not violate the Levene’s test, then results were compared using a two-sample *t*-test. If data violated Levene’s test (*p* < 0.05), non-parametric statistics were performed. A Kruskal–Wallis test was used to determine if model conditions were significant during training. If significant, post-hoc Tukey’s honestly significant difference test was performed to determine where significance occurred (*p* < 0.05). All statistical procedures were performed in MATLAB 2023b.

## 3. Results

### 3.1. Model Validation

The six categories of M-mode image data were analyzed independently for cross-validation analysis using the method outlined in Figure 3. Results showed that the Clinical M-mode performed the best with an R^2^ value of 0.98 ± 0.01 (Figure 4). The next best was the Distributed SMART-US model as the inputs showed an R^2^ of 0.95 ± 0.03. Finally, for force prediction using an individual SMART-US sensor, the results were as follows: SMART-US: VL = 0.85 ± 0.09, SMART-US: MH = 0.82 ± 0.10, SMART-US: RF = 0.85 ± 0.07, SMART-US: VMO = 0.74 ± 0.14. Model validation results violated Levene’s test. Therefore, a Kruskal–Wallis test was used to indicate significant difference between model conditions (*p* < 0.0001). A post hoc Tukey’s test showed that the Clinical M-mode model and the Distributed SMART-US were both significantly different from the SMART-US: VL, SMART-US: MH, SMART-US: RF, SMART-US: VMO (*p* < 0.0001) (Figure 4). There was no significant difference between the Clinical M-mode and Distributed SMART-US models.

### 3.2. Model Testing

**Figure 5:**
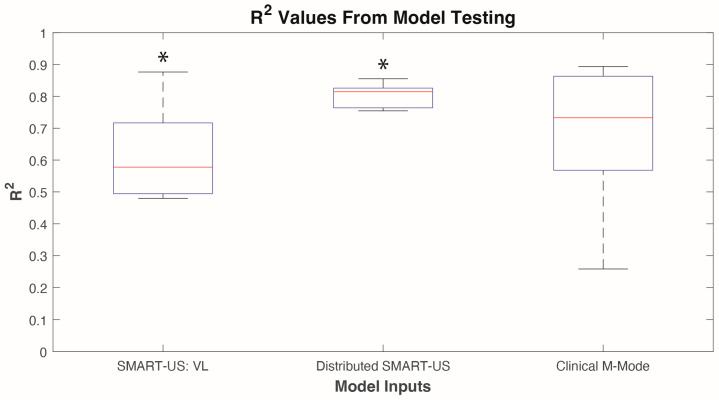
Results of model testing from three different models (n = 5 participants). * *p* < 0.05.

Online testing using the last trial was conducted on the two models that were not significantly different in the cross-validation (i.e., Distributed SMART-US and Clinical M-mode). The SMART-US: VL was also included in model testing, as the sensor was placed in the same location as the Clinical M-mode sensor. Model testing data did not violate Levene’s test (*p* = 0.66) and a two-sampled *t*-test was used to indicate significance between model types. Results showed that the Clinical M-mode model had an average R^2^ of 0.68 ± 0.25, SMART-US: VL model had an average R^2^ of 0.62 ± 0.16, and Distributed SMART-US mode had an average R^2^ of 0.80 ± 0.04 (Figure 5). Two-sample *t*-test indicated that only SMART-US: VL vs. Distributed SMART-US models were statistically significant from each other (*p* < 0.05).

## 4. Discussion

As demonstrated in this research, a novel distributed wearable ultrasound system can accurately predict force during a maximal isometric squat task. Three hypotheses were tested: (1) M-mode ultrasound imaging can be used to predict force during a maximal isometric squat task by utilizing a machine learning algorithm; (2) force predictions from a novel wearable ultrasound system are comparable to those obtained from traditional unportable clinical ultrasound systems; and (3) a novel distributed ultrasound device will have higher prediction accuracy than traditional single anatomical site imaging modalities due to its ability to image multiple muscles that contribute to force production. 

Model validation results indicated that a ridge regression model using Clinical M-mode was able to predict force production during an isometric squat task with an R^2^ value of 0.98 ± 0.01. These results are comparable to previous literature using machine learning models that utilize M-mode imaging metrics to predict force during isometric hand dynamometry tasks [34]. The current study expands upon the previous literature by utilizing a novel distributed imaging system, allowing for analysis of force development from a whole muscle group and focusing on lower body isometric movements. 

Validation results comparing the Distributed SMART-US model and the Clinical M-mode model were not statistically significant, providing evidence that a Distributed SMART-US model can be used as a reliable alternative to predicting force during isometric squats. The Distributed SMART-US model and the Clinical M-mode model were significantly different from the individual SMART-US sensor models. This can be attributed to the Distributed SMART-US model’s multi-sensing modality and the Clinical M-mode model’s higher imaging resolution. 

Testing results also found no statistical significance in model accuracies when comparing force prediction from the Distributed SMART-US and the Clinical M-mode models. Even though it was not statistically significant, the Distributed SMART-US model showed higher precision and robustness across all participants than the Clinical M-mode model, demonstrating its advantage. 

The precision of the Distributed SMART-US model could be attributed to its multi-anatomical imaging nature. Muscles work together to produce a force or movement. When performance of one of the muscles in the group is affected, another muscle will recruit and activate more strongly [53,54]. By having multiple sensors placed over the quadriceps and hamstring, multiple muscle forces in the Distributed SMART-US model can be accounted for, whereas the Clinical M-mode model only examines the force contribution from a single muscle. Additionally, bulky strapping mechanisms, used for transducer placement (Figure 1a), can restrict muscle movement during exercise and influenced the Clinical M-mode model’s variability.

In previous studies, single anatomical site acquisition was identified when comparing sEMG, ultrasound, and dynamometry fatigue assessments [55]. A study conducted by Varol et al. found weak associations among sEMG, ultrasound, and dynamometry systems and identified the necessity to measure all three to provide synergistic information in fatigue assessments [55]. A distributed sensing system, which can provide deep muscle information from multiple anatomical sites, could provide more accurate information about muscle group fatigue during a dynamic task.

sEMG has been used commonly in monitoring muscle fatigue as well as in control paradigms for prosthesis and exoskeletons [7,14,19]. Previous research has shown that, while sEMG may outperform ultrasound-based machine learning models to predict different levels of force in a grip task, ultrasound imaging can be a more robust and accurate measure when muscle fatigue is incorporated into the protocol [56,57]. Additionally, Zhang et al. showed that machine learning models improved in accuracy when ultrasound features were added in addition to sEMG to predict ankle dorsiflexion moment [58]. They theorized that this was due to ultrasound’s ability to directly visualize muscle activity, which is unobtainable in sEMG devices. 

Furthermore, sEMG can monitor multiple anatomical locations simultaneously to probe synergistic muscle activation patterns, though this use case has been cautioned against in previous literature. Tweedle et al. showed that muscle activation measured using ultrasound signals occurring 98 milliseconds before muscle activation was detected by both sEMG and fine wire EMG in the vastus lateralis and biceps brachii [59]. Hence, the use of ultrasound imaging for detection of muscle activation is recommended due to its ability to visualize deep-seated muscle tissue, which can allow for a more accurate interpretation of results. 

As previously discussed, the use of ultrasound technology for monitoring muscle function has increased in recent years. Current research on the implementation of miniaturized wearable ultrasound technology has included multiple use cases ranging from prosthesis control to monitoring physiological signals [37,40,42,48,57].

While our frequency-domain approach to ultrasound is unique in the literature, there are other published works using miniature or wearable A-mode ultrasound with a comparable number of channels. For example, Yang et al. presented a wearable multi-channel A-mode ultrasound system that can be used for in vivo muscle deformation detection and virtual prosthesis control demonstrating a task completion rate of 100% and path efficiency of 93.30% [37]. The device was capable of imaging up to 4 cm at a frame rate of 100 Hz with an overall size of 132 mm × 90 mm × 30 mm. In Jin et al., a 4-channel system capable of imaging up to 8 cm at 90 Hz was used and placed on one muscle. The purpose of the 4 channels was to ensure the muscle interface would always be present in the case of dynamic movement; however, they utilized a commercial pulser stored in a backpack on the subjects during dynamic activity [49]. In addition, Lin et al. developed a wearable ultrasound patch that can be used to continuously monitor physiological signals over a 12-h period [42]. These studies focused on utilizing wearable ultrasound systems for upper body tasks or monitoring physiological signals (i.e., heart rate, blood pressure) at a single location [37,42]. To our knowledge, this is the first study utilizing a novel distributed wearable ultrasound system for lower limb imaging and force prediction during an isometric task. 

Our frequency-domain method that relies on down-mixing of transmit and receive chirps facilitates conversion of signals from MHz to kHz prior to digitization. Therefore, the electronic components and the circuitry in our SMART-US system are similar to those found in FM-radio. Kilohertz data signals post-digitization also makes our approach highly amenable to Bluetooth-based wireless data transfer to portable smart devices. The number of sensors can be readily increased and distributed across the body for simultaneously assessing muscle-level activity at different anatomical sites.

Results from the current research can potentially improve the understanding of complex muscle mechanisms responsible for force output. While this device and methodology may never replace traditional force plate testing protocols, it could be utilized to provide force predictions in austere settings where force plate are unavailable or unusable. In addition, the wearable nature of the presented device means that it can be easily worn during activity and could be incorporated into rehabilitation protocols and workflows in conjunction with traditional force plate testing. This technology enables muscle function to be analyzed in real-time, providing feedback on neuromuscular performance and recovery during activity, which is currently unavailable [60]. 

The incorporation of machine learning is useful for monitoring individual muscle force development and how it relates to overall forces exerted during a task. The potential for this research and SMART-US ranges from rehabilitation to prosthesis and exoskeleton control and can provide biofeedback warnings about compensatory movements that could increase the risk of injury. 

Limitations of the current study included utilization of a single force plate, small sample size of healthy individuals, single anatomical site acquisition of the clinical ultrasound images, testing time and fatiguing exercise protocol. Single force plate collection could have affected machine learning models as force production was the total force produced between both limbs. However, imaging modalities were only placed on the participants’ dominants limb, therefore machine learning models did not consider the non-dominant side when predicting force. Additionally, a fundamental limitation is that machine learning models were subject-specific, and one model cannot be generalized to the whole group. Moreover, validation and testing on different performance tasks are needed to discern the importance of sensor location. This includes additional testing of the clinical ultrasound system on the other muscle groups analyzed by SMART-US, other than the vastus lateralis. By testing the clinical ultrasound system at the different anatomical locations at which the SMART-US sensors where placed, a site-by-site comparison could have been made providing more information on the accuracy of the machine learning model. However, due to the nature of the isometric squat protocol, adding additional imaging locations would have increased collection time and exercise repetitions, fatiguing the participants, which could have influenced results. Additionally, the image resolution of the clinical and SMART-US systems was not compared, and the difference may have impacted feature extraction and quality. Furthermore, clinical transducer pressure and strapping was variable across participants, potentially inhibiting muscle movement during exercise and influencing results.

Future work to address the aforementioned limitations would include: (1) expanding sample size and including different population types, including those who are undergoing late-stage rehabilitation protocols; (2) developing a semi-real-time algorithm that can determine if muscles are engaging or disengaging during isometric tasks; (3) utilizing SMART-US for more complex dynamic performance testing. Additionally, collecting longitudinal data during a rehabilitation period can shed light on how muscle groups react to injury types, locations, and treatments. This type of knowledge can inform clinical decision-making and improve rehabilitation outcomes. 

## 5. Conclusions

In conclusion, this study has demonstrated that a distributed wearable SMART-US system can predict ground reaction force using a linear ridge regression model. This research has the potential to be expanded into understanding muscle function patterns during recovery after musculoskeletal injuries and can potentially help personalize patient care. The overall results of this study prove the feasibility of wearable ultrasound as a force estimation device. The technology presented here has potential to be used in austere environments when traditional force plate testing is unavailable and can provide a more detailed muscle-level analysis of force production during rehabilitation, which is currently unavailable. This device, overall, can create new possibilities and expand the understanding of injury rehabilitation by utilizing information obtained at a muscular level.

## Figures and Tables

**Table 1:** Description of model inputs for machine learning.

Models Inputs	Description
Clinical M-mode	M-mode images from a traditional MSK-US system placed on the dominant vastus lateralis
Distributed SMART-US	M-mode images from all four SMART-US sensors placed on the dominant vastus lateralis, rectus femoris, vastus medialis oblique, and medial hamstring.
SMART-US: VL	M-mode images from SMART-US sensor placed on the dominant vastus lateralis
SMART-US: RF	M-mode images from SMART-US sensor placed on the dominant rectus femoris
SMART-US: VMO	M-mode images from SMART-US sensor placed on the dominant vastus medialis oblique
SMART-US: MH	M-mode images from SMART-US sensor placed on the dominant medial hamstring

## Data Availability

Data are available from the corresponding authors upon reasonable request.
